# Autism and related neurodevelopmental disorders: the many genes involved

**DOI:** 10.1186/1471-2164-15-S2-O22

**Published:** 2014-04-02

**Authors:** Stephen W Scherer

**Affiliations:** 1 The Centre for Applied Genomics, Hospital for Sick Children and McLaughlin Centre, University of Toronto, Canada; 2 Center of Excellence in Genomic Medicine Research, P.O. Box: 80216 Jeddah 21589, King Abdulaziz University, Kingdom of Saudi Arabia

## 

Autism spectrum disorders (ASDs) affect ~1% of the population and are characterized by impairments in social interaction and communication, as well as by repetitive and restricted behaviours. ASDs include mild to severe levels of impairment, with cognitive function ranging from above average to intellectual disability (ID), and are often accompanied by seizures and other medical problems. There is an ~4:1 male to female gender ratio in ASD.

ASDs are highly heritable and genomic studies have revealed that a substantial proportion of ASD risk resides in rare variation of high effect, ranging from chromosome abnormalities and copy number variation (CNV) to single nucleotide variation (SNV). These studies have highlighted a striking degree of genetic heterogeneity, implicating both de novo germline mutation and rare inherited variation in ASD distributed across numerous genes. De novo CNVs are observed in 5%–10% of screened ASD cases, some of which have proven to alter high-risk genes now tested for in the medical diagnostic setting. Exome-sequencing studies estimated another ~6% contribution to ASD, and an additional 5% conferred by rare inherited recessive or X-linked loss-of-function SNV. A genetic overlap between ASD and other neuropsychiatric conditions has also been increasingly recognized.

Interestingly, CNV testing and exome-sequencing have so far yielded mostly non-overlapping genes, which may reflect different mutational mechanisms, though they may still perturb connected biological pathways. While numerous ASD genes have been recognized to date, they only account for a small fraction of the overall estimated heritability, consistent with predictions that there are ~1000 loci underlying ASD and that many causal genes and risk variants remain to be identified.

Here, I will discuss our latest genomic experiments to identify and characterize additional ASD risk genes, and to identify the biological relationships and common pathways they share. Our combined results suggest that rare variants affecting ASD risk in the population collectively encompass hundreds of genes some being influenced by the gender of the mutation carrier. Despite significant heterogeneity, genes group in a relatively small number of interconnected functional networks, particularly related to neuronal signalling/development, synapse function and chromatin regulation. All of these findings, ultimately, contribute to our understanding of the cellular pathways involved in ASD, providing new diagnostic and therapeutic targets.

